# Inheritance of a Ring Chromosome 21 in a Couple Undergoing *In Vitro* Fertilization (IVF): A Case Report

**DOI:** 10.1155/2011/158086

**Published:** 2011-07-31

**Authors:** Roberto L. P. Mazzaschi, Donald R. Love, Ian Hayes, Alice George

**Affiliations:** ^1^Diagnostic Genetics, LabPlus, Auckland City Hospital, P.O. Box 110031, Auckland 1148, New Zealand; ^2^School of Biological Sciences, University of Auckland, Private Bag 92019, Auckland 1142, New Zealand; ^3^Northern Regional Genetic Service, Auckland City Hospital, Private Bag 92024, Auckland 1142, New Zealand

## Abstract

An amniotic fluid sample from an *in vitro* fertilized pregnancy was referred for cytogenetic analysis based on a Down syndrome screening risk of 1 : 21. Routine cytogenetic analysis showed a nonmosaic karyotype of 46,XX,r(21)(p11.2q22.3), with partial monosomy for chromosome 21 due to a ring chromosome replacing one of the normal homologues. Detailed ultrasound scanning for the remainder of the pregnancy did not reveal any unusual findings. Parental bloods showed that the mother was mosaic for the ring 21 with a karyotype of 46,XX,r(21)(p11.2q22.3)/46,XX and the father had an unrelated Robertsonian translocation, with a karyotype of 45,XY,rob(13;14)(q10;q10). Microarray analysis of cultured amniocytes determined the extent of the deletion of chromosome 21 material in the ring. The parents were given genetic counselling, and a phenotypically normal female baby was delivered at term. This case highlights the importance of karyotyping as an initial step in the management of couples referred for *in vitro* fertilization.

## 1. Introduction

Constitutional ring chromosomes are a rare occurrence, for the most part arising spontaneously at an estimated frequency of 1/50,000 among human fetuses [1, 2]. Compared to its normal linear homologue, a ring chromosome is structurally abnormal with the loss of both telomeres. Ring chromosomes are divided into two groups: supernumerary, where the ring is additional to two normal copies of the chromosome, and those cases where one normal homologue has been replaced by a ring equivalent. The former is a partial trisomy for the chromosome involved, while the latter is a partial monosomy for the missing telomeric ends.

Mosaicism for a ring chromosome can also complicate the clinical picture, with a cell line carrying a normal chromosome complement ameliorating the effect of monosomy in the cell line carrying a ring chromosome. The proportions of different cell lines with balanced and unbalanced chromosome arrangements can also vary between different tissues in carriers.

Ring chromosome 21, termed r(21), is a well-documented chromosome rearrangement, with over sixty reported cases [3, 4]. The clinical phenotype of carriers is remarkably variable and depends largely on the extent of the deletion of the telomere of the long arm; Mendelian inheritance and mosaicism of r(21) have also been described [5]. Here, we report a case of familial r(21) in an amniotic fluid sample of a fetus derived by *in vitro* fertilisation (IVF).

## 2. Clinical Report

A routine karyotype request was received on a 15-week gestation amniotic fluid sample from a 40-year-old woman, which was referred based on a nuchal translucency measurement of 3 mm. Her adjusted Down syndrome risk was calculated as 1 : 21. No other information was available apart from a note saying that this was an IVF pregnancy. In addition to conventional karyotyping, rapid trisomy screening using fluorescence *in situ *hybridization (FISH) was also requested.

### 2.1. Fluorescence *in Situ* Hybridisation and Conventional Karyotyping

FISH studies on uncultured interphase cells from the amniotic fluid sample were carried out using an aneuscreen kit according to the manufacturer's instructions (Vysis, Inc.). A preliminary result was issued that was consistent with a female sex complement, with no evidence of trisomy for chromosomes 13, 18, or 21 (data not shown).

**Figure 1:**
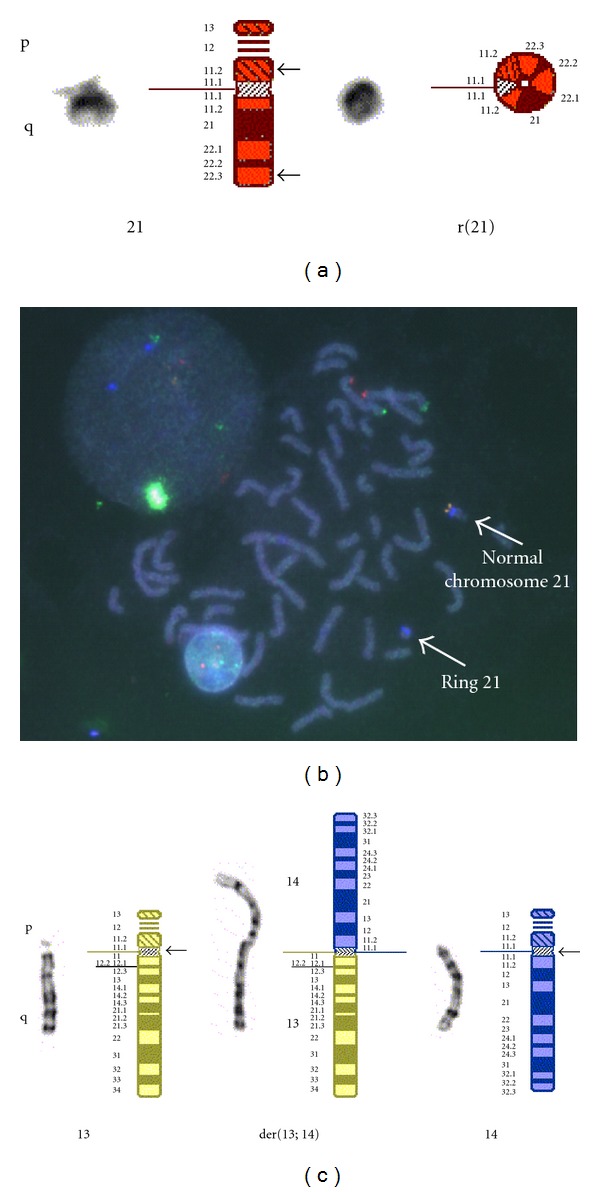
Normal chromosome homologues and relevant rearranged chromosomes in proband and parents; (a) shows the normal and ring chromosomes 21 of the proband; (b) Is a FISH image showing loss of telomeres on the r(21); (c) Shows the Robertsonian translocation chromosome detected in the proband's father.

Routine chromosome analysis was carried out on the amniotic fluid sample using standard techniques and GTG banding. The initial analysis of 15 cells examined from four independent cultures showed a nonmosaic karyotype of 46,XX,r(21)(p11.2q22.3), Figure 1(a). Parental blood samples were requested to determine the origin of the ring chromosome.

An analysis of 50 metaphase cells from the mother's peripheral blood sample showed a female mosaic karyotype of 46,XX,r(21)(p11.2q22.3)[15]/46,XX[35]. FISH analysis using a chromosome 21 subtelomere probe on metaphase preparations of both the mother's peripheral blood and amniotic fluid cells from her pregnancy showed loss of the subtelomeric region on the ring chromosome (Figure 1(b)). Interestingly, cytogenetic analysis of the father's blood sample revealed a 45,XY,rob(13;14)(q10;q10) karyotype, with an apparently balanced Robertsonian translocation between chromosomes 13 and 14 (Figure 1(c)). The rob(13;14) chromosome was not present in the amniotic fluid sample, but has a bearing on the genetic counselling of the parents.

### 2.2. Molecular Karyotyping

**Figure 2:**
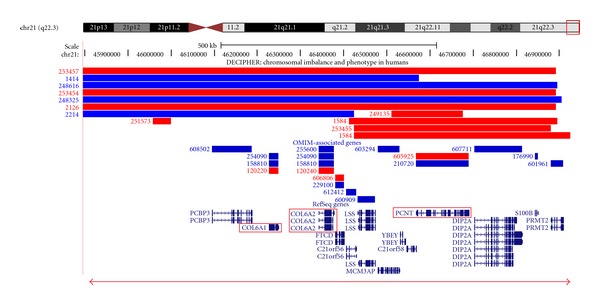
Location and extent of the loss of genomic DNA on the ring chromosome 21. An ideogram of chromosome 21 and the genes that are located within the deleted region of the ring chromosome 21 are shown. Entries in the DECIPHER database (deletions are shown in red, and duplications are shown in blue), OMIM genes, and RefSeq genes are taken from the UCSC genome browser http://genome.ucsc.edu (Human Mar. 2006 (NCBI36/hg18) assembly). The deletion cases in DECIPHER have proximal breakpoints at positions 40.16 Mb (case 253457), 43.99 Mb (case 253454), and 45.35 Mb (case 2126), and that reported by Bertini et al. [4] (not shown above) starts at 43.50 Mb; all four deletions end at 46.89 Mb. The *COL6A1*, *COL6A2,* and *PCNT* genes are shown in red boxes. The horizontal red line with arrow heads denotes the extent of the chromosome 21 loss detected in the case reported here.

DNA was extracted from cultured cells of the amniotic fluid sample, and a molecular karyotype was undertaken to determine the extent of the chromosome 21 loss. Monosomy was detected for a 1138 kb region with the chromosome 21 coordinates 45795806 bp–46933606 bp (Human Mar. 2006 (hg18) assembly, Figure 2). 

The DECIPHER database [6, http://decipher.sanger.ac.uk/] describes three individuals with deletions encompassing the region reported in this case study (253457, 253454, and 2126). They have variable features including isolated clinodactyly, mental retardation, developmental delay, speech delay, cleft lip and palate, strabismus, and hypertelorism, although none of these features are common to all patients. Of the genes that are localised to the deleted region, three appear to be relevant. The first two are *COL6A1* and *COL6A2*, mutations in which are implicated in Bethlem Myopathy (OMIM 158810), which is characterised by proximal muscle weakness, decreased motor capacity, joint contractures, and dystrophic features on muscle biopsy. Frameshift mutations in these genes have been reported for this condition, and nonsense-mediated decay might result in haploinsufficiency in these cases. Importantly, there are no reports in the literature of patients with 21q deletions with a Bethlem myopathy phenotype. The third gene is *PCNT* in which homozygous mutations result in microcephalic osteodysplastic primordial dwarfism type II (MOPD2; OMIM 210720). Interestingly, Rauch et al. [7] found a significant reduction in the mean height of heterozygous MOPD II individuals. The genetic underpinning of the mental retardation and developmental/speech delay in our case remains unclear.

### 2.3. Further Testing

After genetic counselling, the parents opted to continue with the pregnancy. The couple already had a healthy eight-year-old boy, who was subsequently karyotyped and found to be 46,XY. The pregnancy went to term, and we received follow-up chorionic villi, membrane, and blood samples for further cytogenetic analysis. Thirty cells from each of these tissues showed the same nonmosaic ring 21 karyotype as the original amniotic fluid sample. On examination at 6 weeks of age, the infant's head circumference was noted as being 35.5 cm (25th centile), length 55 cm (50th centile), and weight 4.04 kg (50th centile). Clinical examination was normal apart from a left-sided ear tag. Audiology was normal as was a renal ultrasound.

### 2.4. Previous Paternal Testing

A retrospective review of the couple's notes provided additional information. Prior to this current pregnancy, the couple had a period of five years of secondary infertility, which was primarily attributed to a male factor. Semen analysis initially showed oligospermia, with a volume of 4.2 mLs, a count of 9 million sperm/mL, a motility of 5%, and 7% normal forms. A repeat analysis undertaken six months later gave a total sperm count of 6.5 million/mL with only 24% exhibiting “good” mobility, which was considered suitable for intracytoplasmic sperm injection (ICSI) only. Two years later, the couple underwent a cycle of IVF/ICSI. Four eggs were obtained, of which three were fertilised and two were transferred, but a successful pregnancy was not achieved on that occasion. A second cycle of IVF a year later resulted in a single embryo transfer and the current pregnancy.

## 3. Discussion

This case was extremely challenging from a genetic counselling perspective. There was little information in the literature comparing the size of the deletion in the ring 21 and a phenotypic outcome. A report by McGinniss et al. [8] showed that small terminal deletions are usually associated with familial cases and that the phenotypes in these cases tend to be normal. Patients with larger terminal deletions are usually *de novo *cases and exhibit abnormal phenotypes. The interpretation of the cause of the abnormal phenotype in patients with larger deletions is complicated by the fact that these patients tend to be the ones with cells that have lost the ring and/or formed double rings.

The deletion in our patient lies within the boundaries of familial phenotypically normal cases [8]. The discovery of the same ring in the mother should have had a reassuring effect in the counselling of this pregnancy. The mother only had the r(21) in 30% of her blood lymphocytes; the majority of her cells were apparently normal (46,XX). Therefore, any harmful phenotypic effects of such a chromosome rearrangement may well have been ameliorated in the mother. No other tissues were examined in the mother, and so the full extent of her mosaicism remains unknown. 

The mosaicism in the mother meant that no conclusions could be drawn as to a genotype/phenotype correlation in the ongoing pregnancy, and so a molecular karyotype of cultured amniotic fluid cells was undertaken. Interestingly, no familial cases of a ring 21 with a normal phenotype have been studied by microarray analysis. This deficiency might reflect ascertainment bias as the cases with abnormal phenotypes are more likely to have a microarray. 

Decipher and ECARUCA databases suggest that some patients with deletions of a similar size to the one in this pregnancy can exhibit dysmorphism/major organ abnormalities, so this risk cannot be excluded. The ECARUCA database carries one patient (Patient 12) who appears to have a close match to the patient reported here. Patient 12 is attending University and exhibits few facial dysmorphisms, including upslanting palpebral fissures, a broad nasal bridge, and a cleft palate. This patient provides a more reassuring positive outcome for our case study. In addition, unpublished data from the ECARUCA aneuploidy 21 project suggests that the expected degree of intellectual impairment from a deletion of the size found here would be either mild or within normal variance. The future development of the loss of the ring 21 in some cells, or formation of a double ring, cannot be excluded although the absence of this in the mother and the amniocentesis provides some reassurance. The presence of a mildly increased nuchal translucency casts a small degree of concern that the ring 21 chromosome was already giving rise to a phenotypic effect. 

Given the above complicating factors, it was difficult to provide the parents with a clear picture of the likely outcome of the pregnancy. Mild intellectual impairment was the worst likely outcome from a learning perspective. Dysmorphism appears to be more common in cases with larger deletions, but could not be excluded. A detailed ultrasound scan was performed at 22-week gestation and was normal. This made the likelihood of major organ abnormalities less likely, but could not be excluded.

Microarrays can help determine the exact size of a chromosomal imbalance, but the lack of extensive datasets, together with variable phenotypes in comparable cases, means that microarrays may not clarify a phenotypic outcome. This needs to be discussed with the family prior to performing a microarray. 

It should be noted that a 45,XX,-21 karyotype was seen in single cells from 6 different colonies in the amniotic fluid sample, as well as from the follow-up cultured chorionic villus sample taken at term. None of these represented true mosaicism. This demonstrates the phenomenon of dynamic mosaicism; if sister chromatid exchange (SCE) occurs in the ring chromosome during DNA replication, there is a possibility of interlocking or double ring formation. Such structures would be forced to a single pole at cell cleavage, leaving the other daughter cell monosomic for that chromosome. This would explain the observation of single cells with a 45,XX,-21 karyotype in culture. It should also be stressed that no cells were found containing multiple or double rings in any of our cultured tissues. Obviously, a cell containing two stable rings as well as a normal copy of chromosome 21 would be effectively trisomic for that chromosome and have three copies of the Downs critical region [5]. Although the rapid FISH/aneuscreen test could not detect the monosomy caused by the r(21), it did show that there was no evidence of three copies of chromosome 21 (caused by multiple rings or otherwise). Another aspect of dynamic mosaicism is so called “Ring syndrome,” which may have been an added concern in this pregnancy. The continual production of these additional abnormal cells (which may well be nonviable *in vivo*) in dividing tissues can have the net effect of growth retardation *in utero*. 

The surprising additional discovery of an apparently balanced Robertsonian translocation in the father led to a better understanding of possible reproductive difficulties the couple had experienced in the past. This understanding led them to undergo IVF but did not change the clinical picture of the ongoing pregnancy. Robertsonian translocations occur with a population frequency of approximately 1/1000 in newborn surveys, and it is well documented that male carriers of rob(13;14) Robertsonian translocations are at a tenfold increased risk of oligospermia compared to the general population [9]. In addition, the meiotic outcome of paternal uniparental disomy for chromosome 14 among offspring is associated with marked developmental delay, growth retardation, and dysmorphic features [10]. The couple elected not to undertake UPD studies. Despite the cytogenetic obstacles faced by this couple, they already had an apparently healthy, naturally conceived, and chromosomally normal, eight-year-old boy. 

If the couple had been karyotyped at the beginning of their IVF treatment, it is likely that the management of their care would have taken a totally different course, including the possibility of PGD (preimplantation genetic diagnosis). Similarly, the discovery of a ring chromosome 21 in the mother would also have led to very specific genetic counselling, as well as possibly being viewed as a contributory cause to their secondary infertility. 

They had had a previously unsuccessful round of IVF, prior to the case study. If unbalanced forms of either the father's Robertsonian translocation, or the r(21) from the mother, were present in the transferred embryos, these could have been causes of implantation failure. Further family studies should also be carried out for the siblings of both parents. Finally, this case study highlights the value of cytogenetic studies being carried out on couples with a history of reproductive failure, before embarking on more costly IVF treatment. The use of microarrays and FISH merely provided supportive information.
